# Functional characterization and molecular fingerprinting of potential phosphate solubilizing bacterial candidates from Shisham rhizosphere

**DOI:** 10.1038/s41598-023-33217-9

**Published:** 2023-04-28

**Authors:** Samiksha Joshi, Saurabh Gangola, Vandana Jaggi, Manvika Sahgal

**Affiliations:** 1 School of Agriculture, Graphic Era Hill University, Bhimtal, 263136 India; 2 Department of Microbiology, GB Pant University of Agriculture and Technology, Pantnagar, 263145 India

**Keywords:** Microbiology, Molecular biology, Environmental sciences

## Abstract

Phosphate solubilizing bacteria (PSB) are important role players in plant growth promotion. In the present study, we aimed to screen the functionally active phosphate solubilizing bacteria (PSB) associated with *Dalbergia sissoo Roxb.* (Shisham) from different provenances. Screening for phosphate solubilization was done on Pikovskaya's agar, and 18 bacteria positive for the tri-calcium phosphate (Ca_3_(PO_4_)_2_ solubilization showing visible dissolution halo zones were identified. All 18 isolates showed zinc solubilization, indole acetic acid (IAA), siderophore, and hydrogen cyanide (HCN) production. The morphological and biochemical characterization with 16S rDNA gene-based phylogenetic analysis identified bacterial strains as belonging to the genus *Pseudomonas*, *Klebsiella, Streptomyces, Pantoea, Kitasatospora, Micrococcus,* and *Staphylococcus*. Among all the isolates, one of the isolates named L4, from Lacchiwala region was the most efficient P solubilizer with a high P solubilization index (4.75 ± 0.06) and quantitative P solubilization activity (891.38 ± 18.55 μg mL^−1^). The validation of phosphate solubilization activity of PSB isolates was done by amplification of the Pyrroloquinoline quinone (PQQ) genes, *pqqA* and *pqqC*. Based on this study, we have selected the bacterial strains which are efficient phosphate solubilizers and could be economical and eco-friendly in plant growth promotion, disease suppression, as an antioxidant, and for subsequent enhancement of yield.

## Introduction

Shisham (*Dalbergia sissoo* Roxb.) is a nitrogen fixing tree species belonging to the family-Fabaceae. It is similar to nitrogen fixing agricultural crops in forming root nodules through symbiosis with rhizobia and is extensively used for commercial practices^1^. Shisham is an important timber tree, growing throughout sub-Himalayan tract upto 1200 m of altitude^2^. It is a valued tree species, and its global popularity has increased greatly over the past few decades owing to its potent fast growth, multi-purpose uses and nitrogen-fixing ability^3^. Shisham is used for its high-quality timber, fuelwood with various byproducts as well as for its intercropping system to produce maximum yield of forage based farming system^4^. The decrease in productivity of Shisham affects the source of income of rural families and global economy^5^. Wilting is one of the most devastating plant diseases worldwide causing Shisham mortality. Soilborne pathogens are important production constraints leading to reduced growth, yield loss, and threaten adult tree and young tree populations^6^. The declining population of Shisham can be effectively protected by the application of functional bacteria^6,7^.

The rhizosphere represents the intense zone of plant–microbe interaction. Among the microbes bacteria are the most abundant taxonomic group^8^. Rhizospheric bacteria that exhibit plant growth promotion characteristics are known as plant growth promoting rhizobacteria (PGPR). Rhizobacteria promotes growth in plants directly by synthesizing plant growth hormones, enhanced uptake of nutrients or indirectly by inhibiting the phytopathogens attack as well as many other mechanisms^1^. Rhizobacteria promoting plant growth and providing protection from wide range of plant pathogens via several direct and indirect mode of actions are called microbial biological control agents (MBCA). PGPR are potential agents for disease suppression of several phytopathogens and induction of systemic resistance against nematodes and insects via synthesis of antimicrobial metabolites^9^. In addition, some other mechanisms of beneficial bacteria such as competition, interfering with the host immunity to establish a mutualistic association with the host and antagonism can protect plants against pathogen attack^10,11^. Berg and Koskella^12^ reported that beneficial members of plant microbiome can contribute to boost host immune functions. Moreover, immunity of plant may play a major role in determining growth and accommodation of beneficial microbes which further contributes to the association of a stable microbial community inside as well as in their root zone, thus playing a crucial role in regulating variations in microbiota composition^11,13^. However, the microbial composition in rhizospheric region is decided majorly by plant secondary metabolites and root exudates^14^.

Several PGPRs (*Rhizobium*, *Burkholderia*, *Klebsiella*, *Pseudomonas*, *Azotobacter* and *Bacillus*) are reported for N_2_ fixation, P solubilization, siderophore production, zinc solubilization and phytohormone production^14,15^. Bacteria solubilize the insoluble phosphate in medium by oxidation of glucose to gluconic acid or its derivative i.e., 2-ketogluconic acid. The production of acid reduces the soil pH which aids in mineralization of phosphate and makes it available to the plant root^16^. Beneficial effects of P solubilizing bacteria on crops has been evaluated by Raymond^17^. Numerous applications of PSB make it essential to explore their diversity which may further help to design alternative strategies and use these potent strains as bioinoculants. Moreover, community structure is seen to be affected by several factors such as host interaction, fertilizer application, irrigation, and climate^3^. In order to identify endogenous PSB with greater ability to survive under stress conditions and develop as biofertilizers for diverse crops, it is required to learn about the bacterial diversity among them to assess the extent of changes in the bacterial community. The selection of the dominant strains of bacteria involved in P-solubilization that are used as biofertilizers can be aided by knowledge of the molecular diversity of PSB.

Various organic acids viz., gluconic acid, citric acid, malic acid, oxalic acid, fumaric acid, malonic acid, tartaric acid, propionic acid, glyoxylic acid, butyric acid, glutaric acid and adipic acid are reported for phosphate solubilization but among these gluconic acid is the most commonly produced by phosphate solubilizing bacteria^18,19^. Production of gluconic acid mainly occurs in bacteria with the help of enzyme glucose dehydrogenase encoded by gcd (glucose dehydrogenase) gene under direct oxidation pathway^20^. A cofactor pyrroloquinoline quinone encoded by pqq operon consisting of six core genes (*pqqA-F*) is required for the effective functioning of GDH enzyme^21^. The cloning and expression of genes involved in biosynthesis of PQQ showed the importance of gluconic acid and its derivative 2-ketogluconic acid production in phosphate solubilization^22^. Sonnenburg and Sonnenburg^23^ suggested that signature genes primarily involved in pqq biosynthesis pathway are *pqqA*, *pqqC*, *pqqD*, and *pqqE* which were recognized by gene knockout experiment. The majority of identified pqq genes in bacterial isolates belongs to α, β, γ class of proteobacteria and primarily present in gram-negative bacteria^24^. The commonly found bacteria genera with PQQ gene belongs to *Acinetobacter, Azotobacter, Beijerinckia, Bradyrhizobium, Burkholderia, Erwinia, Gluconoacetobacter, Klebsiella, Gluconobacter, Methylobacillus, Methylobacterium, Mycobacterium, Pseudomonas, Rhizobium, Streptomyces,* and *Xanthomonas*^24^. Growth conditions such as high glucose concentration as a carbon source and high insoluble phosphate level significantly affect the biosynthesis of glucose dehydrogenase and PQQ level^21^. The characterization of PSB colonizing rhizosphere of Shisham trees and their effects on plant growth under stress condition remains under explored. Hence it is necessary to investigate the effect of P solubilizing bacterial diversity on soil health, and mechanism involved in the rhizospheric region.

Therefore, in the current study we aimed to explore the rhizosphere of Shisham trees from various unexplored soils and screen the most effective P-solubilizing bacteria in mitigating environmental stress conditions.

The primary aim of this study was to:

1. find the optimal P-solubilizing bacteria that are most effective under various environmental and growth conditions by screening the Shisham of different unexplored soils.
2. functional and molecular characterization of isolated PSB strains to explore the biodiversity among different rhizospheric regions of Shisham.
3. validate the corresponding mechanisms and genes involved in P-solubilization.

## Material and methods

### Soil sampling

Soil samples were collected from three different rhizospheric regions of Shisham forests located at three sites: Pantnagar (29.0222° N latitude, 79.4908°E longitude), Lachhiwala (30.2230°N latitude, 78.0766° E longitude) and Tanakpur (29.0722°N latitude, 80.1066° E longitude) regions in India. The three sites represent different agroecological zones and niches, each diversified with distinct vegetation cover, soil, and other natural resources. The Shisham trees in Lachhiwala and Tanakpur forest were healthy but the Shisham trees in Pantnagar forest were diseased. Furthermore, from each forest region, three trees were identified for rhizospheric soil sample collection within the range of 1–10 m. The samples were collected in triplicate from rhizospheric soil (15 cm depth) of Shisham trees of a forest region during winter season. The samples were pooled together to generate a representative composite sample and transferred in sterilized soil sampling plastic bags (zip lock) to the laboratory and kept at − 20 °C till further analysis.

### Soil physico-chemical characteristics

Soil samples were air dried for soil physico-chemical analysis. Soil physico-chemical analysis included determination of soil pH, electrical conductivity, total organic carbon (TOC), total nitrogen (TN), available potassium (AK) and trace elements such as Fe and Zn^3,25^. To verify the results statistically, One-Way Analysis of Variance (ANOVA) was used at level of p < 0.05 using SPSS software.

### Soil enzymatic assays

Each soil sample was analyzed for their significant contribution of microbial community (soil microbial enzymes) in rhizospheric region with the help of spectrophotometer. The exact concentration of the analyzed soil enzymes was determined by plotting a standard curve. All soil microbial enzymatic assays were performed in triplicates. Dehydrogenase activity was determined as reported by Thalmann^26^. Fluorescein diacetate (FDA) activity was determined according to Inbar et al.^27^. Alkaline and acid phosphomonoesterases activity was assayed according to a method of Tabatabai and Bremner^28^. Urease activity in soil was determined as given by Kandeler and Gerber^29^.

### Soil microbial enumeration

Enumeration of bacteria in rhizospheric soil (total aerobic bacterial count) was enumerated through serial dilution pour plating on Angles’s medium^30^ whereas for phosphate solubilizing bacteria, Pikovskaya medium was used^31^. The bacterial population in per gram of soil was determined by counting and expressing as colony forming unit (CFU) after 2–3 days of incubation at 30 ± 1 °C. Both the media were supplemented with 100 mgL^–1^ of cycloheximide to inhibit fungal growth^30^.

### Selection of rhizobacterial isolates based on biochemical and plant growth promotion traits

The biochemical characterization of the bacterial isolates was conducted this includes amylase, urease, nitrate reductase, lipase, xylanase, protease, pectinase, and catalase activity. In vitro PGP traits of the rhizobacterial isolates were assessed for production of siderophore, indoleacetic acid (IAA), ammonia, hydrogen cyanide (HCN) and solubilization of zinc. For all these biochemical and functional traits analysis protocols were followed described by Joshi et al.^30^.

### Phosphate solubilizing efficiency of phosphate solubilizers

Phosphate solubilizing bacteria were isolated using serial dilution and pour plate technique on Pikovskaya’s medium (PK medium). To provide optimum growth conditions the inoculated plates were incubated at 28 ± 2 °C for 3–4 days within the incubator. The bacterial colonies surrounded with halo zones were picked and restreaked to obtain pure cultures. All pure cultures were spot inoculated on Pikovskaya medium and incubated at 30 °C for 48 h. Halo zones surrounding the colonies were measured. Solubilizing efficiency (SE) and solubilization index (SI) of PSB isolates were also calculated^30,32^.

$${\text{Solubilization Efficiency }}\left( {{\text{SE}}} \right) \, = \frac{{\text{Diameter of bacterial growth}}}{{\text{Diameter of clear zone}}} \times 100$$

$${\text{Solubilization Index }}\left( {{\text{SI}}} \right) \, = \frac{{{\text{Diameter of bacterial growth }} + {\text{ Diameter of clear zone}}}}{{\text{Diameter of colony}}}$$

### Quantitative estimation of phosphorous

Selected bacterial cultures were subjected to transfer in 25 mL National Botanical Research Institute's phosphate growth medium (NBRIP: glucose (10 g L^−1^), calcium phosphate (5 g L^−1^), magnesium chloride hexahydrate (5 g L^−1^), magnesium sulfate heptahydrate (0.25 g L^−1^), potassium chloride (0.2 g L^−1^), ammonium sulfate (0.1 g L^−1^)) for 72 h at 28 ± 1 °C and 120 rpm. After completion of successive growth period the bacterial isolates were centrifuged for 15 min at 5000 rpm. Supernatant (1 mL) was taken in a test tube and added with 60% perchloric acid (0.4 mL); molybdate solution: 2.5% ammonium molybdate in 5 N H_2_SO_4_ (0.4 mL); colouring reagent: 10 mL of 5% sodium bisulphate, 20% sodium sulphite, 25 g 1-amino-2-naphthol-4-sulphonic acid (0.2 mL), and triple distilled water or TDW (4 mL) subsequently. After that the test tubes were incubated for 30 min at room temperature. The appearance and intensity of blue color exhibit the total concentration of phosphorus and measured the absorbance at 640 nm^33^.

### Molecular characterization, identification, and phylogenetic analysis

Genomic DNA of all 18 isolates were extracted using alkaline lysis method^34^ and the purity was checked in agarose gel. Amplification of 16S rDNA was done using template DNA of all 18 bacterial isolates recovered from different provenances of Shisham. Forward primer GM3f (5ʹ TACCTTGTTGTTACGACTT3ʹ) and reverse primer GM4r (5ʹTACCTTGTTACGACTT3ʹ) were used for amplification of 16S rDNA gene. PCR product was electrophoresed in 1.0% agarose gel at 80 mA for 1 h along with λ DNA/EcoRI/HindIII double digest ladder^3^. Further the purified 16S rDNA amplicon products were sent to Biotech Centre UDSC, New Delhi for sequence analysis. The obtained nucleotide sequences were processed for homology using BLASTn through EzBioCloud's database (https://www.ezbiocloud.net/identify)^35^. All the sequences were aligned with MEGA7 (Molecular Evolutionary Genetic Analysis version 7.0) software for constructing a phylogenetic tree^36^.

### Fingerprinting of selected bacterial isolates

Purified 16S rDNA amplicon of each 18 isolate was digested with three tetra cutter restriction endonucleases namely *Msp*I,* Alu*I, and *BsuR*I. The digestion reaction was set in a reaction mixture of 25 µL, which included 20 µL amplicon and reaction mixture with 1X assay buffer for enzyme, 1U/reaction of each restriction endonuclease *Msp*I, *Alu*I, and Fast digest *BsuR*I. For digestion with *Msp*I and *Alu*I reaction mixture was kept at 37 °C for 2 h and *BsuR*I fast digest for 5 min. Thereafter the enzymatic reaction was inactivated by adding loading dye and kept at − 20 °C. The product of restriction digestion was analyzed on 2.5% agarose gel electrophoresed at 60 V. The band pattern was visualized under UV Gel documentation system^37^.

### Amplification of *pqqA* and *pqqC* gene

The bacterial genomic DNA of selected isolates was subjected to amplification using Gen Amp PCR System 9700 (Applied Biosystems) in a 20 μL volume. The primers used for *pqqA* gene were forward primer *pqq*A-F: 5ʹATGTGGACCAAACCTGCATAC3ʹ and reverse primer *pqq*A-R:5ʹGCGGTTAGCGAAGTACATGGT3ʹ, while the primer set for *pqqC* gene were forward primer *pqq*C-F:5ʹATTACCCTGCAGCACTACAC3ʹ and reverse primer *pqq*C-R:5ʹ CCAGAGGATATCCAGCTTGAAC 3ʹ. The composition of reagents was:10X Assay Buffer (1×), MgCl_2_ (0.5 mM), dNTPs (200 µM), Taq polymerase (1U), Forward and reverse primer (0.3 μM), Template DNA (50 ng). For the amplification of 2 PQQ genes the reaction conditions were as follows: Initial denaturation 94 °C for 5 min (1 cycle); denaturation 94 °C for 30 s (30 cycles); annealing 50 °C for 30 s; extension 72 °C for 1 min; and final extension 72 °C for 10 min (1 cycle). The presence of amplified fragments was checked on 2.0% (^w^/_v_) Agarose gel with 50 bp DNA ladder^37^.

### Statistical analysis

The experimental data (qualitative and quantitative) were statistically processed using t-test (Cochran and approx t-test). All results were expressed as mean ± SEM. F values for which *p* < 0.05 were considered significant^30^.

## Results

### Soil physico-chemical analysis

Soil physico-chemical analysis was performed to assess the soil nutrient status and health. The analysis of macro and micro-nutrient contents along with some other important parameters (soil type, pH and electrical conductivity) of Shisham rhizospheric soil from three different provenances are presented in Table 1. The soil texture was silty loam in Lachhiwala and Tanakpur region whereas it was silty clay loam in Pantnagar. Soil pH in Pantnagar soil was 6.85 which was comparatively higher than Lachhiwala and Tanakpur (6.00 and 6.12) respectively. Electrical conductivity was found to be 0.11 dS m^−1^ for Lachhiwala, 0.14 dS m^−1^ for Tanakpur and 0.13 dS m^−1^ for Pantnagar. Total organic carbon in Pantnagar, Lachhiwala and Tanakpur was 42,750 kg hac^−1^, 19,500 kg hac^−1^ and 25,000 kg hac^−1^ respectively. Further available phosphorus in soil was highest in Lacchiwala (56.48 kg hac^−1^) as compared to Pantnagar (37.86 kg hac^−1^) and Tanakpur (46.87 kg hac^−1^). Total nitrogen (TN) in Pantnagar, Lachhiwala and Tanakpur was 137.98 kg hac^−1^, 163.07 kg hac^−1^ and 100.35 kg hac^−1^ respectively while soil potassium was 505.34 kg hac^−1^, 434.11 kg hac^−1^, and 520.12 kg hac^−1^ respectively. The iron (22.6 kg hac^−1^) and zinc (11 kg hac^−1^) content was highest in Tanakpur soil as compared to the Lachhiwala (Fe: 12.5 kg hac^−1^; Zn: 9.3 kg hac^−1^) and Pantnagar soil (Fe: 11 kg hac^−1^; Zn: 0.2 kg hac^−1^) (Table 1). Soil nutrient properties were analysed statistically. The ANOVA (p < 0.05) results revealed highly significant differences between soil nutrient values at Lachhiwala, Tanakpur and Pantnagar.

**Table 1:** Soil physico-chemical properties in three different Shisham provenances.

Properties	Pantnagar	Lachhiwala		Tanakpur
Soil pH	6.85	6.00		6.12
Electrical conductivity	0.13	0.11		0.14
Soil type	Silty clay loam	Silty loam		Silty loam
Carbon	42,750 kg hac^−1^	19,500 kg hac^−1^		25,000 kg hac^−1^
Phosphorus (available phosphorous)	37.86 kg hac^−1^	56.48 kg hac^−1^		46.87 kg hac^−1^
Potassium	505.34 kg hac^−1^	434.11 kg hac^−1^		520.12 kg hac^−1^
Nitrogen	137.98 kg hac^−1^	163.07 kg hac^−1^		100.35 kg hac^−1^
Iron	11 kg hac^−1^	12.5 kg hac^−1^		22.6 kg hac^−1^
Zinc	0.2 kg hac^−1^	9.3 kg hac^−1^		11 kg hac^−1^

Each value is the mean of three replicates. Data was analysed statistically at the 5% (p < 0.05) level of significance.

### Soil enzymatic activities

Alkaline phosphatase, acid phosphatase, fluorescein diacetate, dehydrogenase and urease activities of Shisham rhizospheric soils from Shisham forests at three different location was done. Alkaline phosphatase activity ranged from 442.8 µg PNP g^−1^ h^−1^ at Tanakpur to 1196.2 µg PNP g^−1^ h^−1^ at Lachhiwala. The highest activity of acid phosphatase enzyme was found to be 1109.6 µg PNP g^−1^ h^−1^ in Lachhiwala followed by Tanakpur (654.5 µg PNP g^−1^ h^−1^) and Pantnagar (574.8 µg PNP g^−1^ h^−1^). FDA (fluorescein diacetate) activity in Lachhiwala, Tanakpur and Pantnagar was 291.2 µg fluorescein g^−1^ h^−1^, 372.6 µg fluorescein g^−1^ h^−1^ and 325 µg fluorescein g^−1^ h^−1^ respectively. Dehydrogenase enzyme levels were two-fold higher in case of Tanakpur forest (4300 µg TPF g^−1^ h^−1^) as compared to Lachhiwala forest (1880 µg TPF g^−1^ h^−1^) while least activity was reported in the case of Pantnagar forest (1770 µg TPF g^−1^ h^−1^). The maximum urease activity was observed in the Shisham rhizosphere soil from Lachiwala forest (241 µg NH_4_^+^ g^−1^ h^−1^) followed by Pantnagar forest (192.25 µg NH_4_^+^ g^−1^ h^−1^). The minimum urease activity was observed in rhizosphere soil from Tanakpur forest (65.78 µg NH_4_^+^ g^−1^ h^−1^). There was a significant difference (p < 0.05) between the enzyme activities of Shisham rhizosphere soils from three provenances (Table 2).

**Table 2:** Enzyme activities of rhizospheric soil of Shisham at Pantnagar (P), Lachhiwala (L) and Tanakpur (T).

	Locations	Acid phosphatase, µg PNP g^−1^ h^−1^	Alkaline Phosphatase, µg PNP g^−1^ h^−1^	Fluorescein diacetate, µg florecein g^−1^ h^−1^	Dehydrogenase, µg TPF g^−1^ h^−1^	Urease, µg N g^−1^ h^−1^
Enzyme activity	P	574.8 ± 1.15	613.04 ± 0.83	325 ± 0.57	1770 ± 1.15	192.25 ± 0.67
	L	1109 ± 0.66	1196.23 ± 0.63	291.2 ± 1.15	1880 ± 1.15	241 ± 0.56
	T	654.5 ± 0.30	442.8 ± 0.33	372.6 ± 0.57	4300 ± 0.57	65.78 ± 0.65

Each value is the mean of three replicates. Data was analysed statistically at the 5% (p < 0.05) level of significance.

### Soil microbial enumeration

Total population as enumerated on Angle’s medium in Shisham rhizospheric soil of Tanakpur, Lacchiwala and Pantnagar was 2.76 × 10^4^, 1.87 × 10^4^ and 1.96 × 10^4^ cfu g^−1^ of soil. However, count of phosphorus solubilizing bacteria was 1.20 × 10^4^ cfu g^−1^, 1.55 × 10^4^ cfu g^−1^ and 1.06 × 10^4^ cfu g^−1^ soil at Tanakpur, Lachhiwala and Pantnagar respectively. Name of the selected PSB bacterial isolates were coded according to their native rhizospheric region from different forest (Table S1). Bacterial morphological characteristics were also observed^37^ (Table S2).

### Solubilizing efficiency of P solubilizer

**Figure 1:**
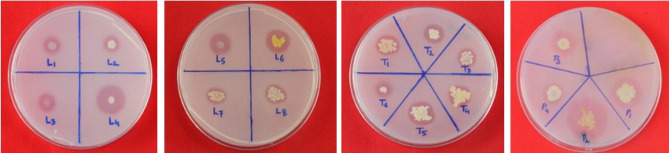
Qualitative efficiency of PSB bacterial isolates depicting halo zone around bacterial colonies on Pikovskaya agar plate.

Overall, 18 PSBs, eight from Lacchiwala, four from Pantnagar and six from Tanakpur were recovered on Pikovaskya agar plates from Shisham rhizospheric soil of different provenances (Fig. 1). All eighteen bacterial isolates exhibited zone of solubilization in the range 1.16 to 4.75 cm on pikovaskya agar plates (Fig. S1). The isolates from Lachhiwala provenance depicted higher phosphate solubilising index as compared to Tanakpur and Pantnagar. Highest P solubilising index (PSI) was detected in L4 and lowest in T4 (Table 3). Bacteria-mediated phosphorous solubilization was quantified by following Fiske and Subbarow (1925) method. Out of the eighteen bacterial isolates, L4 solubilized highest amount of phosphorus (891.38 µg mL^−1^) and T4 (285.78 µg mL^−1^) solubilized lowest amount of phosphorus (Fig. S2). The solubilizing index of PSBs as detected on Pikovaskya agar plates positively correlated with amount of P solubilized in NBRIP liquid medium.

**Table 3:** In vitro qualitative and quantitative estimation of tricalcium phosphate solubilization by bacterial isolates.

S. no	Strain id	P solubilization index* (cm)	P solubilisation (μg mL^−1^)
1	L1	2.75 ± 0.02	590.63 ± 12.57
2	L2	2.50 ± 0.03	506.25 ± 0.79
3	L3	2.75 ± 0.05	559.32 ± 10.76
4	L4	4.75 ± 0.06	891.38 ± 18.55
5	L5	3.50 ± 0.03	721.27 ± 10.51
6	L6	3.75 ± 0.06	762.10 ± 7.5
7	L7	2.60 ± 0.04	530.75 ± 12.15
8	L8	2.80 ± 0.07	616.48 ± 7.05
9	P1	2.50 ± 0.005	510.33 ± 7.17
10	P2	4.00 ± 0.07	850.56 ± 14.16
11	P3	2.85 ± 0.06	639.62 ± 4.66
12	P4	1.85 ± 0.01	476.31 ± 4.46
13	T1	2.60 ± 0.01	537.55 ± 6.01
14	T2	2.60 ± 0.03	523.94 ± 2.99
15	T3	1.71 ± 0.04	340.22 ± 1.39
16	T4	1.16 ± 0.01	285.78 ± 3.27
17	T5	2.00 ± 0.01	421.87 ± 6.36
18	T6	2.50 ± 0.04	559.32 ± 6.98

*Values are the mean of triplicates with standard error of mean.

### Functional characterization of PSB recovered from Shisham rhizosphere

Selected PSB strains were screened for various enzyme activities and plant growth promotory properties. All PSBs exhibited one or more of enzymes amylase, urease, nitrate reductase, lipase, xylanase, protease, pectinase and catalase activity (Fig. S3). Among the eighteen isolates, four isolates such as L7, L8, T3 and T5 were positive for amylase production. Urease test was found positive for L4, P2, T2 and T6. All the isolates except L4, T1, T3, T4, T5 and T6 exhibited nitrate reduction. Out of eighteen PSBs, five; L7, L8, P2, T3 and T5 were positive for lipase activity. Only eight isolates, L7, L8, P1, P4, T1, T3, T4 and T5 were positive for xylanase production. Five isolates from Lachhiwala (L1, L2, L5, L7 and L8), one each from Pantnagar (P2) and Tanakpur (T5) were positive for protease production as a halo zone was observed around bacterial growth on skim milk agar plates. Six out of eighteen isolates L1, L5, P1, P3, P4 and T1 were positive for pectinase enzyme production. Except L6, T1 and T3 all the isolates were able to produce catalase as production of gas bubbles and effervescence was observed after addition of drops of H_2_O_2_.

Amongst 18 PSBs, seven isolates were able to solubilize Zinc. Zinc solubilization efficiency was highest in L3, L5, P2 and T2 and lowest in L4, P3 and P4. Five isolates were positive for siderophore production. Orange halos were maximum in L7, L8, T1, T3 and minimum in L1. IAA production was maximum in L4, P3, T1, T2 and T4 and least in L1, L5, L6, L7, L8, P1, P4, T3 and T5. All the isolates except P2 were negative for HCN production. Ammonia production in peptone water marked by color change from yellow to orange was found positive for all isolates except L6, L7, T1 and T3. Hence, all bacterial isolates exhibited multiple PGP traits along with inorganic P solubilization (Fig. S4; Table S3).

### Molecular characterization, identification and phylogenetic analysis

PCR amplification of 16S rDNA gene region of all eighteen PSB isolates recovered from Shisham rhizosphere of different provenances, resulted in a distinct band of 1492 bp in the agarose gel (Fig. S5). Bacterial isolates were identified by comparison of 16S rDNA sequences with reference strains using BLASTn programme. Out of eighteen isolates seven were identified within genus *Pseudomonas*. Out of these seven, 3 isolates were from Lachhiwala (L1, L3 and L5) whereas four were from Pantnagar (P1, P2, P3 and P4). Four isolates were identified as *Streptomyces sp.* (L6, L7, T3 and T5)*,* two each as *Klebsiella sp.* (L4 and T2) and *Staphylococcus sp.* (L2 and T6)*,* and one each as *Pantoea sp*. (L8), *Kitasatospora sp*. (T1) and *Micrococcus sp*. (T4).

**Figure 2:**
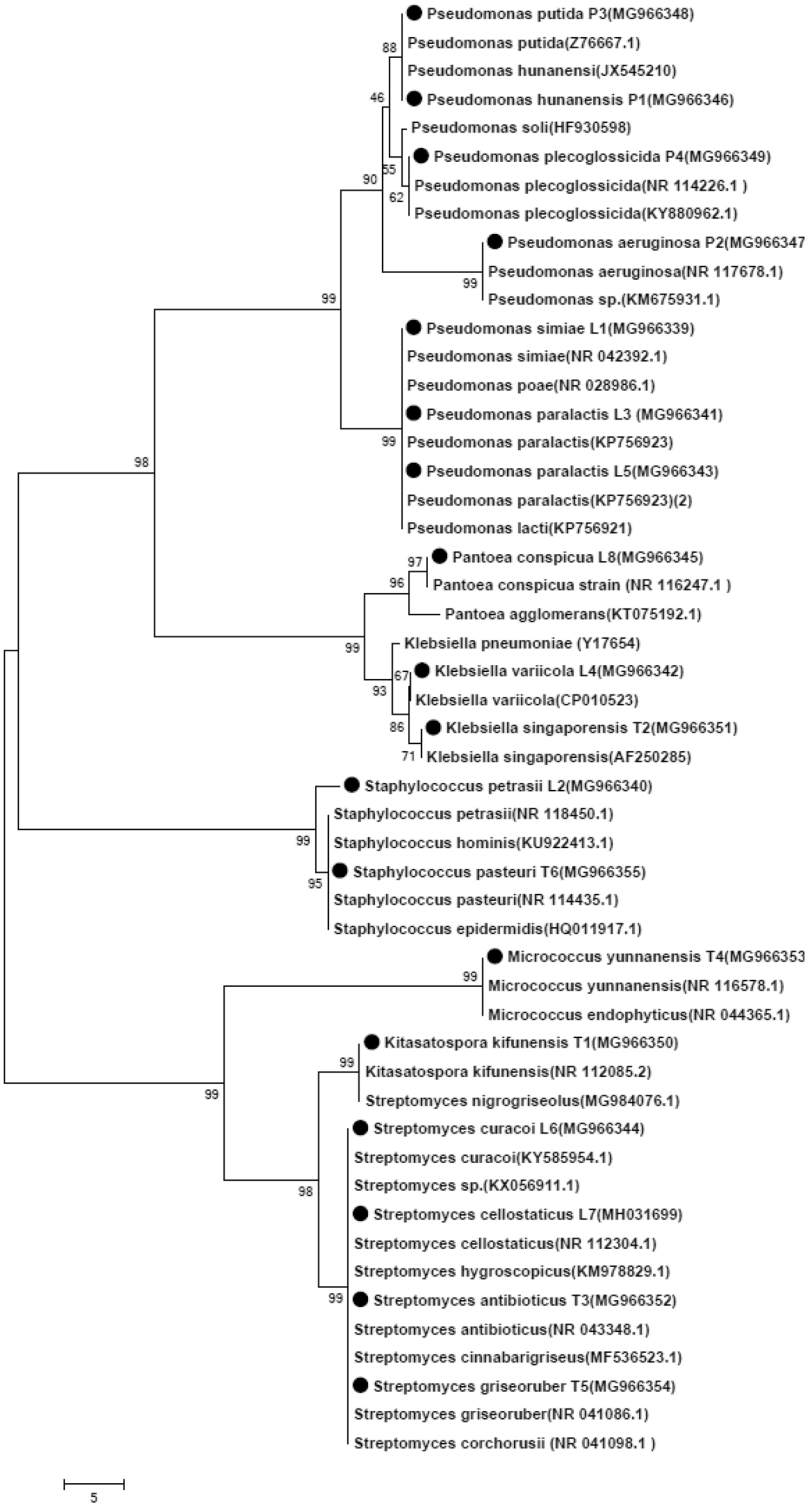
The neighbor joining tree based on 16 SrDNA sequences of bacterial isolates associated with Shisham rhizosphere from three provenances. Tree topology was obtained after 1000 runs. The bootstrap values are indicated at the nodes. Scale bar represents 5 nucleotide substitutions per site. The strains with bold circle are from this study.

All eighteen strains were identified as belonging to 7 genera distributed across three phyla: Proteobacteria, Actinobacteria and Firmicutes. The genera identified were: *Pseudomonas*, *Klebsiella, Streptomyces, Pantoea, Kitasatospora, Micrococcus* and *Staphylococcus* (Fig. 2; Table S4). Seven strains were identified as L1 (98.14% similarity to *Pseudomonas simiae* strain NR 042392.1), L3 and L5 (99.16% similarity to *Pseudomonas paralactis* strain KP756923), P1 (98.89% similarity to *Pseudomonas hunanensis* strain JX545210), P2 (97% similarity to *Pseudomonas aeruginosa* strain NR 117678.1), P3 (98.14% similarity to *Pseudomonas putida* strain Z76667.1) and P4 (98.42% similarity to *Pseudomonas plecoglossicida* strain NR 114226.1). Strain L8 was identified as *Pantoea sp.* (96.83% similarity to *Pantoea conspicua* strain NR 116247.1). Two strains L4 and T2 were identified as *Klebsiella sp*. (99.51% similarity to *Klebsiella variicola* strain CP010523 and 96.37% similarity to *Klebsiella singaporensis* strain AF250285). Strain L2 was assigned to (97.98%) *Staphylococcus petrasii* (NR 118450.1) and T6 to *Staphylococcus pasteuri* (NR 114435.1). Isolates belonging to phylum Actinobacteria were clustered together which includes T4 (98.0% similarity to *Micrococcus yunnanensis* strain NR 116578.1), T1 (93.86% similarity to *Kitasatospora kifunensis* strain NR 112085.2), L6 (87% similarity to *Streptomyces curacoi* strain KY585954.1), L7 (95% similarity to *Streptomyces cellostaticus* strain NR 112304.1), T3 (94.22% similarity to *Streptomyces antibioticus* strain NR 043348.1), T5 (97.92% similarity to *Streptomyces griseoruber* strain NR 041086.1). The 16S rDNA sequences of all eighteen isolates are deposited in NCBI GenBank under accession numbers MG966339-MG966355 (Table S4).

### DNA fingerprinting of selected bacterial isolates

Based on Amplified ribosomal DNA (rDNA) restriction analysis (ARDRA) profiles and morphological characters, isolates were selected and taxonomically identified. After the restriction of amplified 16S rDNA with endonucleases generated 100–1000 bp fragment of DNA. Restriction enzyme *AluI* generated 2–4 well resolved bands of 700 bp to 100 bp in all eighteen isolates. Endonuclease *AluI* resolved all 18 strains into eight different genotypes (Fig. S6a). The restriction pattern of amplified 16S rDNA region with restriction enzyme *BsuI* resulted in 2 to 4 well resolved bands in a range from 1000 to 100 bp. The restriction with *Bsu I* resolved all 18 strains into six different genotypes (Fig. S6b). The restriction profiles obtained with *Msp*I enzyme resulted in one to three well resolved bands in a region from 200 to 600 bp (Fig. S6c). All eighteen isolates were distinguished into eight genotypes.

### Combined UPGMA dendrogram based on DNA fingerprint profiles

An unweighted pair group means average (UPGMA) dendrogram calculating Jaccard’s coefficient was constructed based on analysis of the ARDRA profile of 16S rDNA region with *Alu*I, *Bsu*I and *Msp*I through NTSYSpc version 2.0 software^37^. Restriction profile was interpreted on the basis of bands developed. Similar banding patterns obtained after combination of the three independent digestions were grouped. The isolates depicted higher polymorphism with *Alu*I and *Msp*I as compared to *Bsu*I. Eight different restriction patterns were obtained with *Alu*I and *Msp*I whereas six with *Bsu*I.

Phylogenetic relationship within gram negative and gram-positive isolates were revealed by UPGMA clustering of isolates separately. In a UPGMA cluster based on RFLP with *Alu*I, *BsuI* and *Msp*I, all gram-negative strains grouped into two major clusters A and B (Fig. S7a). Cluster A included five isolates L1, L3, L5, P1 and P3. The cluster A was further divided into two subclusters. Subcluster, I included L1, L3 and L5 and subcluster II grouped P1 and P3. L3 and L5 in subcluster I exhibited 100% similarity and was related to L1 at a distance of 0.80 on Jaccard’s scale. Cluster B included the remaining strains P4 and P2 related at a distance of 0.60 Jaccard’s scale. For gram positive bacteria a separate dendogram was constructed (Fig. S7b). Majority of gram-positive isolates were placed in a single cluster which was further divided into two subclusters at 0.80 on Jaccard’s scale. Subcluster I included two isolates L6 and L7 whereas subcluster II included T3 and T5. Isolate T4 was placed singly on an outlying branch at a distance of 0.60 on Jaccard’s scale. Isolate T1 was distantly (0.35 on Jaccard’s scale) related to all the other strains.

### Amplification of *pqqA* and *pqqC* genes

**Figure 3:**
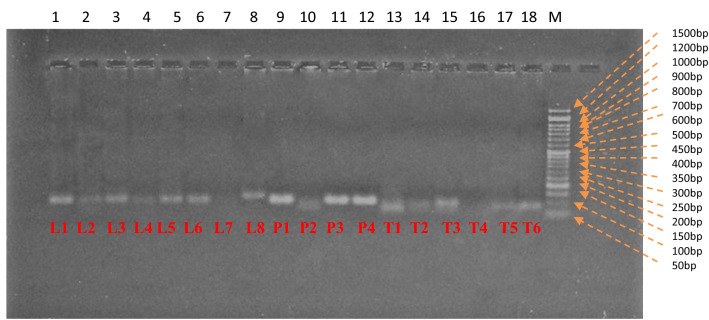
PCR amplicons of partial *pqqC* gene fragment. Lanes: 1. L1; 2. L2; 3. L3; 4. L4; 5. L5; 6. L6; 7. L7; 8. L8; 9. P1;10.P2;11.P3;12.P4;13.T1;14.T2;15.T3;16.T4;17.T5;18.T6 Lanes: M denotes 50 bp DNA ladder.

**Figure 4:**
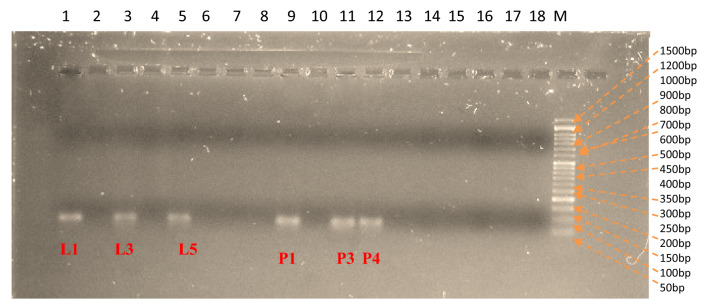
PCR amplicons of *pqqA* gene fragment. Lanes: 1, 3, 5, 9, 11 and 12 represent the amplification of *pqqA* gene from different PSB strains such as L1, L3, L5, P1, P3 and P4 respectively. Lanes: M denotes 50 bp DNA ladder.

To confirm the conserved genomic region (*pqqA* and *pqqC*) for gluconic acid formation, PQQ gene amplification was done with the help of designed primer. Out of eighteen only sixteen bacterial isolates showed positive amplification for *pqqC* gene (82 bp band) whereas six bacterial isolates namely L1, L3, L5, P1, P3 and P4 showed positive amplification of *pqqA* gene (72 bp band) (Figs. 3, 4). All six isolates with positive amplification for both *pqqC* and *pqqA* genes suggests that they possess two crucial genes of PQQ biosynthesis pathway.

## Discussion

Among microorganisms, bacteria play an important role in biogeochemical cycling. Bacteria solubilize the insoluble organic and inorganic phosphates in the soil, which makes P available to plant roots and is considered the most eco-friendly and economic method^38^. PSB are well known for disease suppression by synthesizing pathogen inhibitory compounds as well as enhancing the plant immune response. Hence the aim of this study was to identify the PSB bacteria which suppress plant disease and enhance the plant growth, bringing dual benefits.

Soil of Pantnagar was reported to be silty clay loam with high pH, high carbon, low phosphorus, and low micronutrients (Fe and Zn) content in comparison with other two samples. At a time of sampling, it was observed that rate of mortality in Shisham trees was maximum in Pantnagar soil as compared to others. The reason behind the Shisham mortality in Pantnagar may be the deficiency of micro and macronutrients in soil. Micronutrients are essential for proper functioning of plants as well as to promote growth of beneficial microbes in rhizospheric region^39^. Inadequate amount of micronutrients in soil directly affects the metabolic capacity of plants which further directly affects the tolerance towards biotic and abiotic stress^40^. Macronutrient and micronutrient deficiency in soil affects the yield in crops and plants, invite disease and resist their propagation^41,42^. Hence, low nutrient status (low P, Fe, Zn) in soil of Pantnagar might be associated with disease incidence and spread. Correlation analysis showed that the values of P solubilizing index and the amount of soluble phosphorus in liquid NBRIP medium shared a highly significant relationship (t value = 15.30069) which indicates that the strains with the highest potential to solubilize Ca_3_(PO_4_)_2_ in liquid media were the same as the ones that exhibited the greatest halos. Moreover, slightly high pH of soil could also be the cause for mortality in Pantnagar Shisham forest. Higher soil pH hinders the availability of phosphorous to the plants and alter biological, geological, and chemical environment of soil which leads to disease in plants^43^. Soil enzyme activities and nutrient status are closely related. Soil organic carbon (SOC), phosphorus, nitrogen, potassium and other essential micronutrients significantly affect the activities of the soil enzymes^44^. In the present study Fluorescein diacetate (FDA) and Dehydrogenase activity correlated with culturable microbial population or respiratory metabolism^45^. The dehydrogenase and FDA activities were higher in Shisham rhizosphere from Tanakpur where the aerobic bacterial population was also highest. Soil phosphatase activity is pH sensitive, depending on the number and diversity of soil resident microflora^46^. The acid phosphatase, alkaline phosphatase, and urease activities were higher in Shisham rhizosphere from Lachhiwala which is due to the measure of total microbial population. Pathogens are encouraged to colonise the rhizosphere by increasing carbon levels, whereas helpful bacteria may do so if there are more nutrients available. Hence it indicates that not the individual C and nutrient content but the ratio that affects the rhizosphere microbiome which ultimately alters the soil enzyme status. Organic phosphate is solubilized by group of phosphatase enzymes like acid and alkaline phosphatases, phytases, and nucleotidases^47^. Among which the extracellular acid and alkaline phosphatase play a key role in solubilization. In the terrestrial ecosystem the acid phosphatase is primary synthesized by plant roots and microbial action whereas the alkaline phosphatase is synthesized by microbes^48^. Li et al.,^49^ studied the role of acidic and alkaline phosphatase in subalpine forest region and found that alkaline phosphatase actively participates rather than the acid phosphatase in mineralization and solubilization of phosphorus further making it available to plant roots.

The present study investigated that the different environmental abiotic factors and total organic carbon content of the soil at different provenances could significantly affect PSB population in the rhizospheric region. Microbial population density at rhizospheric region depends on several factors such as physico-chemical property of soil, water potential of soil, change in soil pH, partial pressure of oxygen and chemical composition of plant exudation^50^. Microbial enzymes such as amylase, xylanase, lipase, pectinase, and protease were found actively involved in organic matter decomposition, plant growth promotion and are important in the disease suppression^51,52^. Bacterial genus such as *Pseudomonas, Micrococcus, Paenibacillus, Streptococcus, Curtobacterium, Chryseobacterium* are reported to produce hydrolytic enzymes which degrade the cell wall of pathogenic organisms^52^.

Out of eighteen isolates recovered in the present study, seven isolates were positive for Zn solubilization. Production of organic acids is the prominent mechanism for Zn solubilization by rhizobacteria^53^. Out of 18 isolates, five isolates exhibited yellow to orange halo zone on CAS amended nutrient agar plates for siderophore production. Siderophores may enhance plant growth by mobilizing metal cations including Fe and Cu^54^ as well as indirectly stimulate P solubilization and disease suppression^55,56^. Siderophore positive PGPRs scavenge Fe^3+^ from complex compounds under iron starvation condition and thus indirectly release P in soil^18^. Moreover, they deprive phytopathogen from iron and hence lead to disease suppression^57^.

In the present study, fourteen isolates were potent IAA producers. IAA production by bacteria enhances root growth which leads to increased nutrient uptake in plants^3^. The ability of IAA production by microbes varies among different species and is also affected by availability of substrate, culture conditions and stage of growth^58^. HCN is also reported to play crucial role in disease suppression^59^. Ammonia promotes plant growth by providing N to plants and suppressing plant pathogen^60^.

Isolated bacterial strains were related to genus *Streptomyces, Pseudomonas*, *Klebsiella, Staphylococcus, Kitasatospora, Pantoea,* and *Micrococcus.* Several members within these genera are identified for exhibiting plant growth promoting ability, P solubilizing and biocontrol properties for example: *Pantoea, Pseudomonas* and *Streptomyces*^61^, *Klebsiella* and *Micrococcus*^62,63^, *Kitasatospora* reported for resistance to pest attack and growth promotion in Teak (*Tectonagrandis*), which is a valuable tree species^64^.

Sixteen bacterial isolates showed positive amplification for 82 bp *pqqC* gene whereas six for 72 bp *pqqA* gene. The bacterial isolates that exhibited amplicon for *pqqA* gene were also positive for *pqqC* gene, this suggests that they possess two crucial genes of PQQ biosynthesis pathway. PQQ operon (*pqqA-pqqF*) organize differently in different PSB isolates such as in PQQ operon of *Acinetobacter calcoaceticus*, the *pqqF* gene is absent^65,66^. While in *P. fluorescens* B16, the PQQ operon was composed of 11 genes namely, *pqqA*, *B*, *C*, *D*, *E*, *F*, *H*, *I*, *J*, *K*, and *pqqM*^67^. Hence the presence of *pqqA* and *pqqC* gene in bacterial isolates could be prominent candidate for solubilization of insoluble phosphate. Presence of *pqqA*, *pqqC, pqqD* and *pqqE* genes are prerequisite for P solubilization in PSB isolates^23^. Gene *pqqA* consists of 22 amino acids, a peptide of glutamic acid and tyrosine which serve carbon and nitrogen for PQQ biosysnthesis^65,68^. The *pqqC* gene encodes the pyrroloquinoline quinone synthase C (PqqC), which catalyzes the conversion of 3a-(2-amino-2-carboxy-ethyl)-4,5-dioxo-4,5,6,7,8,9-hexahydroquinoline-7,9-dicarboxylic acid to pyrroloquinoline quinone^69,70^. Therefore we can conclude that the selected bacterial isolates might be following gluconic acid mediated mechanism for solubilization of insoluble P in soil. *pqqC* is ubiquitous in *Pseudomonas* species^71^. High PQQ-producing bacteria have been identified in bacteria of diverse genera, including *Mycobacterium, Acinetobacter, Hyphomicrobium, Gluconobacter, Klebsiella, Polyporus, Ancylobacter, Pseudomonas, Xanthobacter, Methylobacillus, Paracoccus, Methylophilus, Methylobacterium, Thiobacillus* and *Methylovorus*^72^. In the present study, there were several strains in which there was no amplification of *pqqA* and *pqqC* genes. However, they were solubilizing phosphorus on pikovaskya medium. The possible reason is that these strains might be solubilizing phosphate via secretion of organic acids other than gluconic acid such as isovaleric acid, lactic acid, isobutyric acid, glycolic acid, acetic acid, oxalic acid, succinic acid and malonic acid. Bacteria like *E. coli* JM109 (genetically modified), *Synechococcus* PCC7942 (phosphoenol pyruvate carboxylase (*ppc*)); *Serratia marcescens* and *Pseudomonas cepacia* (*gabY*) solubilize P by other than PQQ pathway or gene^73–77^. Therefore, our finding concludes that the deficiency of nutrients and excess availability of carbon and high pH invite the pathogenic microorganisms which is the main cause of wilt in Pantnagar soil. Most of the selected bacterial strains were previously reported for P solubilization. Mechanism of P solubilization through signature genes such as *pqqA* and *pqqC* has been reported for the first time in Shisham forest region.

## Conclusion

In this study we found that the nature of soil and their native microbial community play a crucial role for plant growth and protection. To resolve the problem of mortality in forest soil it is necessary to analyze the physicochemical and biological properties of soil. The deficiency of macronutrients, micronutrients, alteration in soil pH and soil enzymes may lead to invite different kinds of plant disease and plant pathogen. The finding and enrichment of the best PGPR bacterial strains could minimize the mortality of Shisham trees and help to enhance the biodiversity. Amplification of phosphate solubilizing gene (*pqqA* and *pqqC*) in bacterial strain provides strong evidence for the mechanism of phosphate solubilization and their potent solubilizing efficiency. Hence our findings suggested that the bioformulation of bacterial isolates could mitigate the phosphate deficiency and promote plant yield directly as well as indirectly.

## Supplementary Information

Supplementary Information.

## Data Availability

The 16S rDNA sequences of all eighteen isolates are deposited in NCBI GenBank under accession numbers MG966339-MG966355 (https://www.ncbi.nlm.nih.gov/nuccore/). The bacterial isolates and their accession number is also given in the supplementary file.
