# Supplementary material for: Functional characterization and molecular fingerprinting of potential phosphate solubilizing bacterial candidates from Shisham rhizosphere
Source: Sci Rep. 2023 Apr 28;13:7003. doi: 10.1038/s41598-023-33217-9 (PMC10147649; doi:10.1038/s41598-023-33217-9)
Supplement: Supplementary file 1 — Supplementary Information. [file 41598_2023_33217_MOESM1_ESM.pdf]

SUPPLEMENTARY FILE

Functional characterization and molecular fingerprinting of potential phosphate solubilizing bacterial candidates from Shisham rhizosphere

Samiksha Joshi[1], Saurabh Gangola[1], Vandana Jaggi[2], Manvika Sahgal[2*]

[1]School of Agriculture, Graphic Era Hill University Bhimtal, 263136, India

[2]Department of Microbiology, GB Pant University of Agriculture and Technology, Pantnagar, 263145, India

Correspondence: sahgal.manvika@gmail.com

Fig S1: Graphical representation of qualitative estimation for tricalcium phosphate solubilization by bacterial isolates.

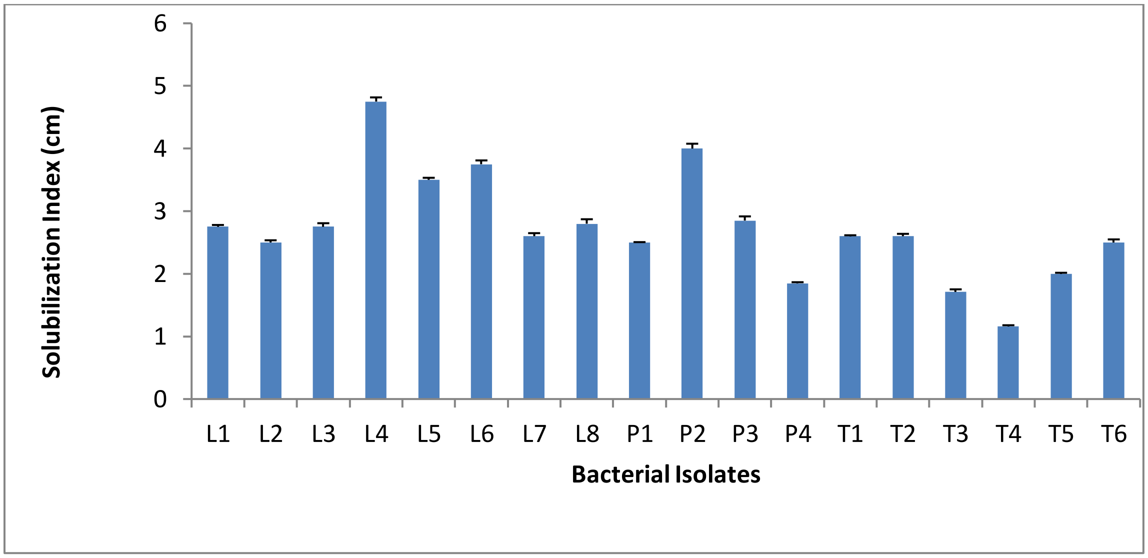

Fig S2: Graphical representation of quantitative estimation for tricalcium phosphate solubilization by bacterial isolates in NBRIP broth medium (pH 7.0) at 30˚C

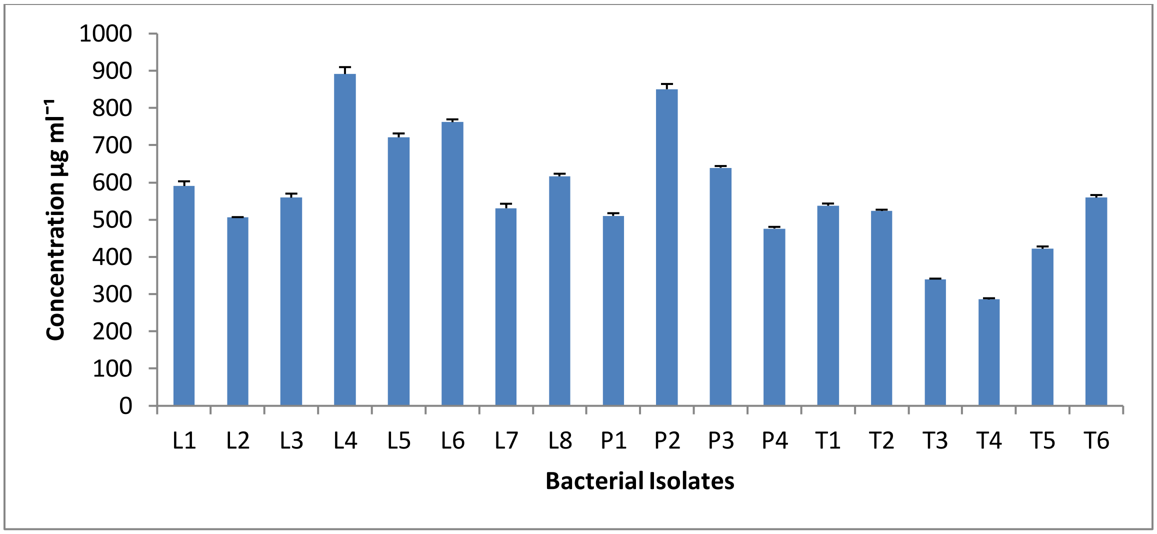

Fig S3: Enzymes and metabolites produced by isolates(a) Amylase (b) Urease(c) Nitrate reductase (d) Lipase (e) Xylanase (f) Protease (g) Pectinase

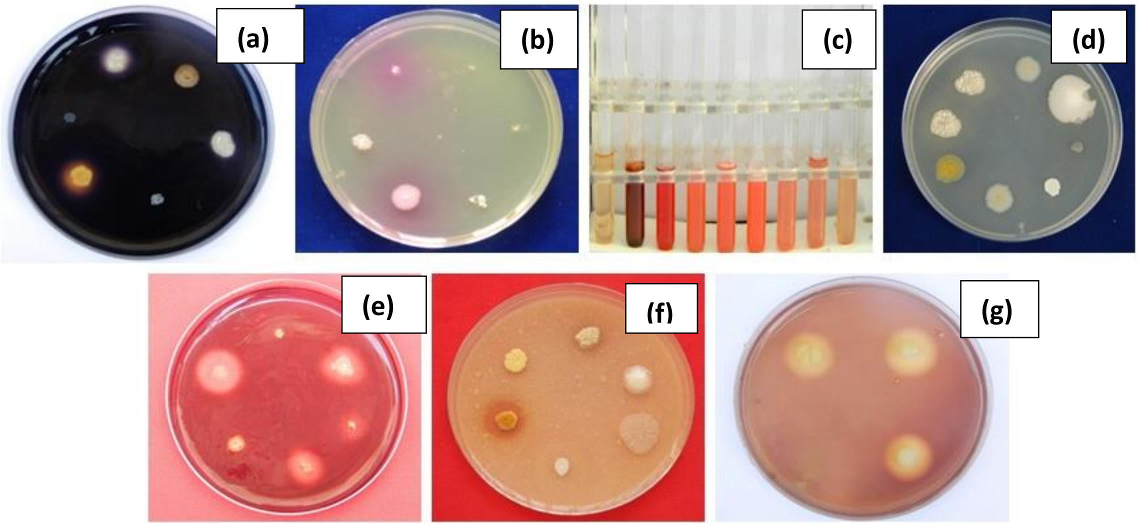

Fig S4: Plant growth promoting properties of isolates(a) Zinc solubilisation(b)Siderophore production (c)HCN production (d) IAA production (e)Ammonia production

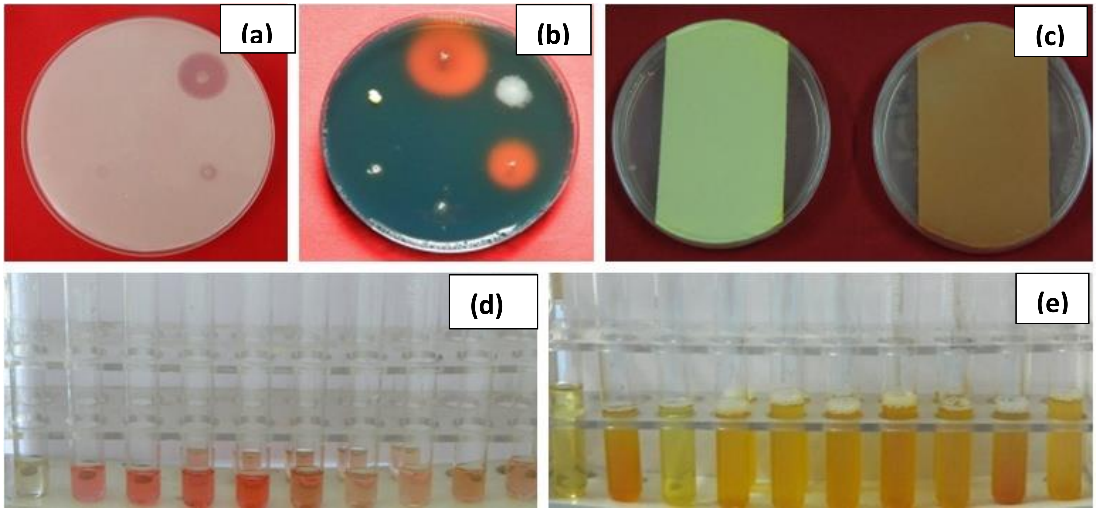

Fig S5: **16SrRNA amplification of the bacterial cultures**Lanes:1. L1; 2. L2; 3. L3; 4. L4; 5. L5; 6. L6; 7. L7; 8. L8; 9. P1;10. P2;11.P3;12.P413.T1;14.T2;15.T3;16.T4;17.T5;18.T6 lane 19= M= Lambda DNA/Eco RI/Hind III Double digest ladder.

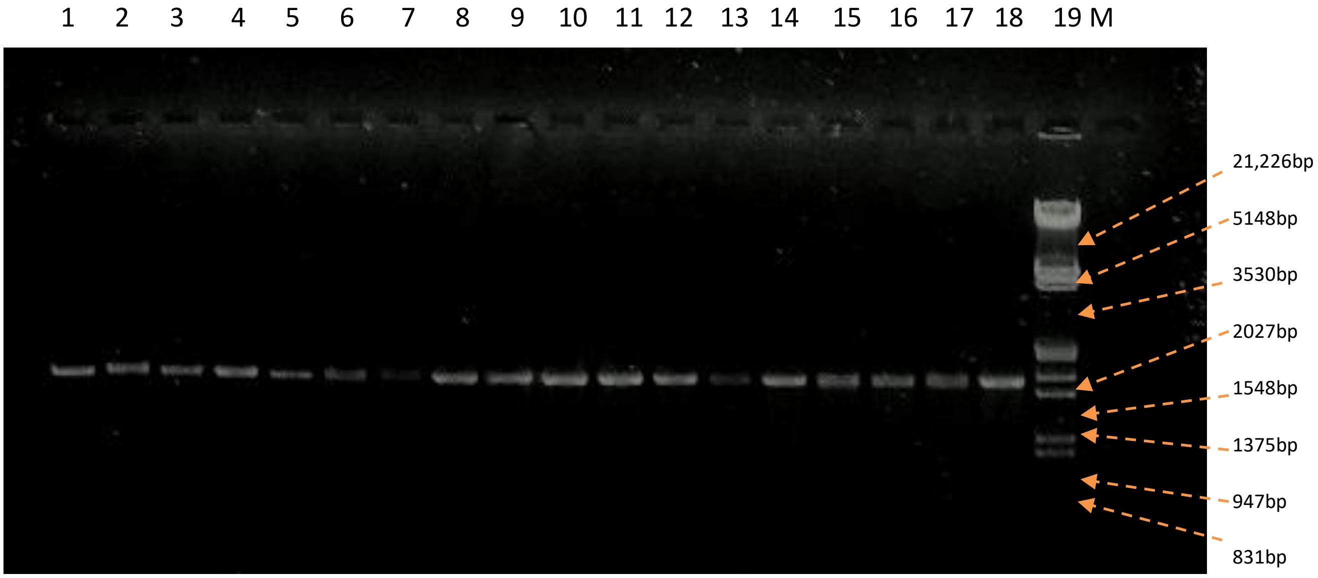

Fig S6: Restriction profile of 16S rDNA using (a) ***Alu*****I, (b)** ***BsuR*****I and (c)** ***Msp*****I. Lane 1-18= Isolates, lane 19= M= molecular weight marker and stepup 100 bp ladder**

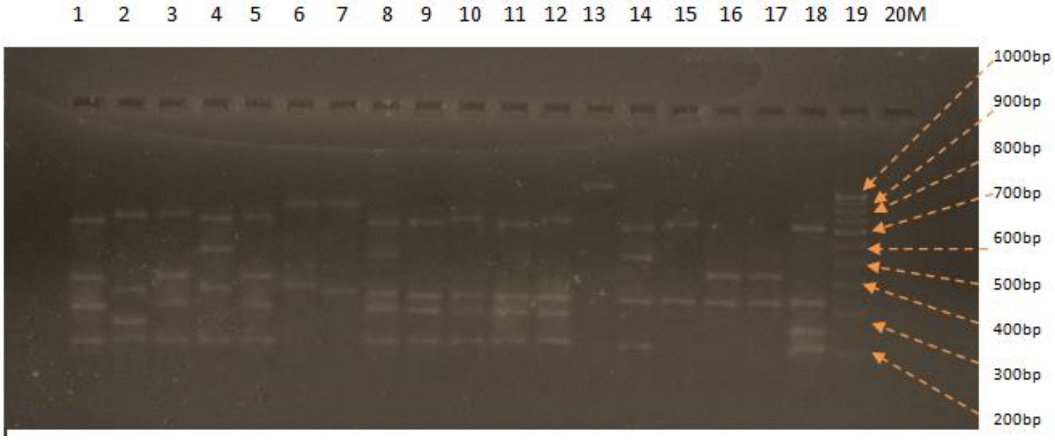

(a)

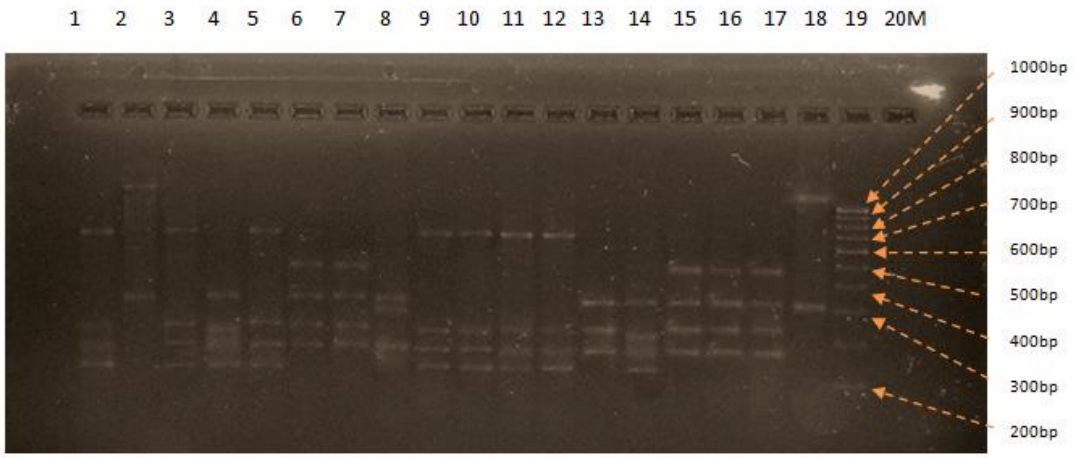

(b)

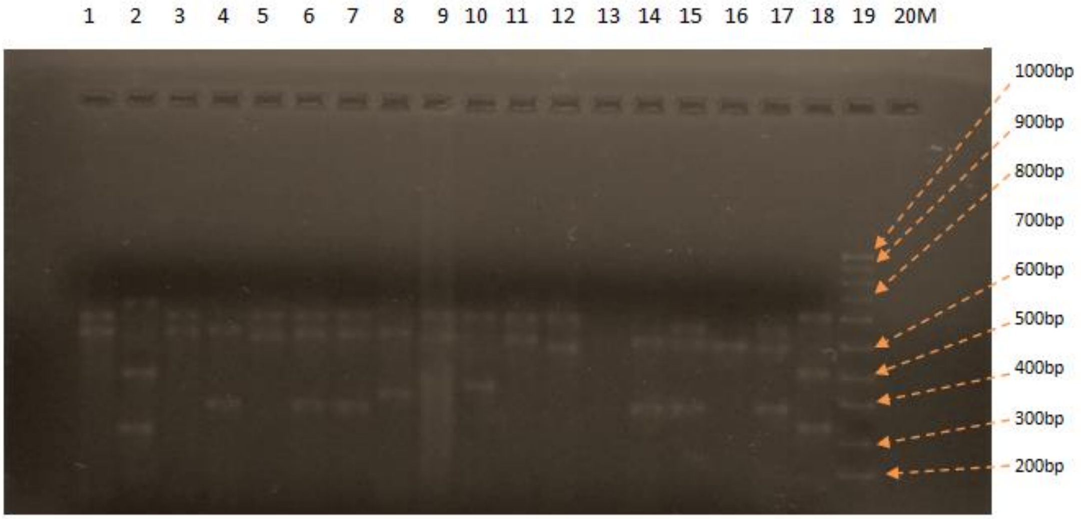

(c)

Fig S7: Combined UPGMA dendrogram of 16S rDNA region of isolates on the basis of ARDRA with *Alu*I, *BsuR*I and *Msp*I

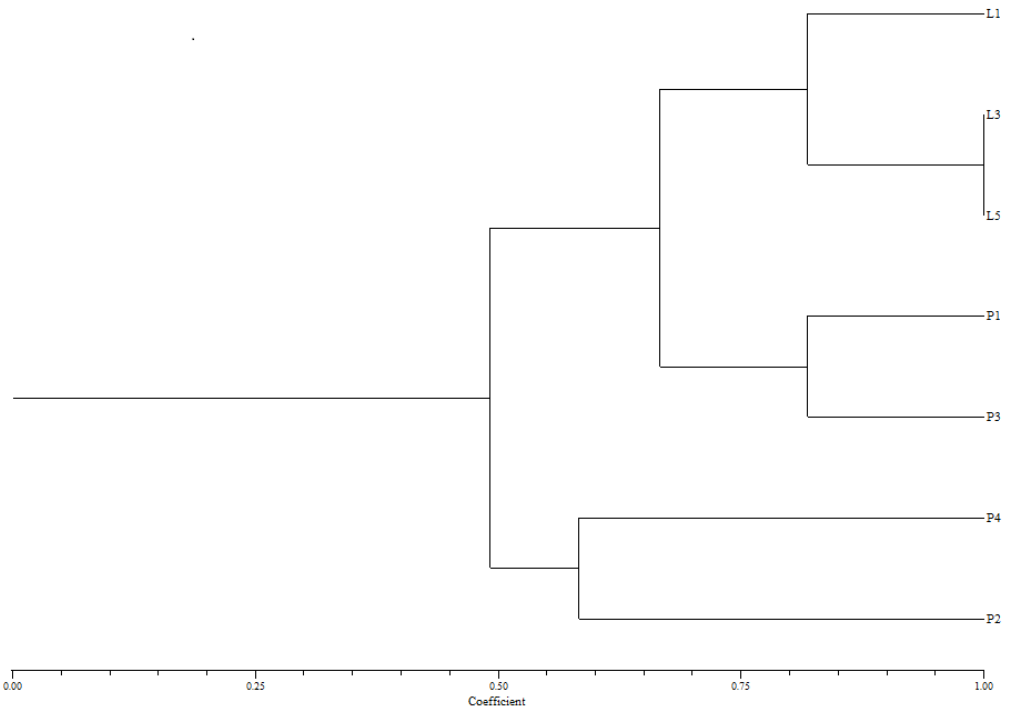
L1
L3
L5
P1
P3
P4
P2
0.00
0.25
0.50
0.75
1.00
Coefficient

(a)

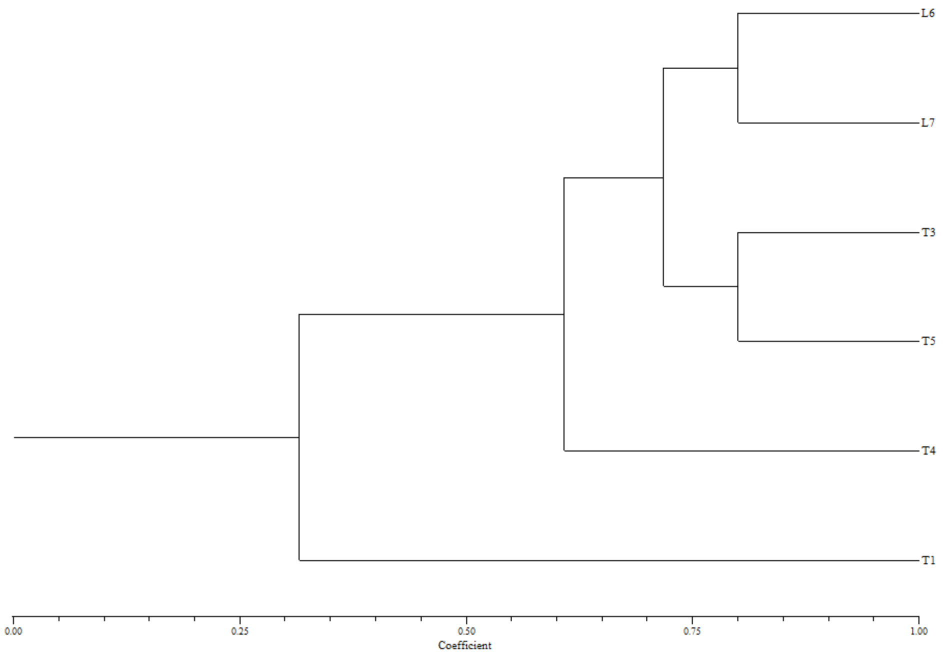
L6
L7
T3
T5
T4
T1
0.00
0.25
0.50
0.75
1.00
Coefficient

(b)

Table S1: Description of sites from where isolates have been recovered

S.no.	Location	Sample no.	Isolate code
1	Lachhiwala	1	L1
2	Lachhiwala	2	L2
3	Lachhiwala	3	L3
4	Lachhiwala	4	L4
5	Lachhiwala	5	L5
6	Lachhiwala	6	L6
7	Lachhiwala	7	L7
8	Lachhiwala	8	L8
9	Pantnagar	9	P1
10	Pantnagar	10	P2
11	Pantnagar	11	P3
12	Pantnagar	12	P4
13	Tanakpur	13	T1
14	Tanakpur	14	T2
15	Tanakpur	15	T3
16	Tanakpur	16	T4
17	Tanakpur	17	T5
18	Tanakpur	18	T6

Table S2: Cultural characteristics of the Bacterial isolates under study

S. No.	Isolate I.D.	Gram Reaction	Cell Morphology
1	L1	Negative	Small rods
2	L2	Positive	Cocci
3	L3	Negative	Small rods
4	L4	Negative	Small rods
5	L5	Negative	Small rods
6	L6	Positive	Filamentous
7	L7	Positive	Filamentous
8	L8	Negative	Small rods
9	P1	Negative	Small rods
10	P2	Negative	Small rods
11	P3	Negative	Small rods
12	P4	Negative	Small rods
13	T1	Positive	Filamentous

14	T2	Negative	Small rods
15	T3	Positive	Filamentous
16	T4	Positive	Coccii
17	T5	Positive	Filamentous
18	T6	Positive	Cocci

Table S3: PGPR properties of phosphorus solubilizing bacteria from three *Dalbergia sissoo* provenances.

Geographical Region	Strains	Zinc solubilisation			Siderophore production			Indole acetic acid production			HCN production			Ammonia production		
		-	+	++	-	+	++	-	+	++	-	+	++	-	+	++
Lachhiwala	L1	■				■			■		■					■
	L2	■			■			■			■				■	
	L3			■	■			■			■					■
	L4		■		■					■	■					■
	L5			■	■				■		■					■
	L6	■			■				■		■			■		
	L7	■					■		■		■			■		
	L8	■					■		■		■				■	
Pantnagar	P1	■			■				■		■					■
	P2			■	■			■				■				■
	P3		■		■					■	■					■
	P4		■		■				■		■					■
Tanakpur	T1	■					■			■	■			■		
	T2			■	■					■	■					■
	T3	■					■		■		■			■		
	T4	■			■					■	■				■	
	T5	■			■				■		■				■	
	T6	■			■			■			■				■	

Table S4: Molecular characterization of the bacterial cultures based upon 16S rDNA sequences

Strain	Isolate	Percent Similarity	NCBIGen Bank Accession no.
L1	*Pseudomonas simiae*	98.14%	MG966339
L2	*Staphylococcus petrasii*	97.98%	MG966340
L3	*Pseudomonas paralactis*	99.16%	MG966341
L4	*Klebsiella variicola*	99.51%	MG966342
L5	*Pseudomonas paralactis*	99.17%	MG966343
L6	*Streptomyces curacoi*	87.00%	MG966344
L7	*Streptomyces cellostaticus*	95.00%	MH031699
L8	*Pantoea conspicua*	96.83%	MG966345
P1	*Pseudomonas hunanensis*	98.89%	MG966346
P2	*Pseudomonas aeruginosa*	97.00%	MG966347
P3	*Pseudomonas putida*	97.00%	MG966348

P4	*Pseudomonas plecoglossicida*	98.42%	MG966349
T1	*Kitasatospora kifunensis*	93.86%	MG966350
T2	*Klebsiella singaporensis*	96.37%	MG966351
T3	*Streptomyces antibioticus*	94.22%	MG966352
T4	*Micrococcus yunnanensis*	98.00%	MG966353
T5	*Streptomyces griseoruber*	97.92%	MG966354
T6	*Staphylococcus pasteuri*	98.20%	MG966355
